# clustComp, a bioconductor package for the comparison of clustering results

**DOI:** 10.1093/bioinformatics/btx532

**Published:** 2017-08-23

**Authors:** Aurora Torrente, Alvis Brazma

**Affiliations:** 1 Departamento de Ciencia e Ingeniería de Materiales e Ingeniería Química, Instituto Gregorio Millán, Universidad Carlos III de Madrid, Leganés, Spain; 2 European Molecular Biology Laboratory, European Bioinformatics Institute (EMBL-EBI), Wellcome Trust Genome Campus, Hinxton, UK

## Abstract

**Summary:**

clustComp is an open source Bioconductor package that implements different techniques for the comparison of two gene expression clustering results. These include flat versus flat and hierarchical versus flat comparisons. The visualization of the similarities is provided by means of a bipartite graph, whose layout is heuristically optimized. Its flexibility allows a suitable visualization for both small and large datasets.

**Availability and implementation:**

The package is available at http://bioconductor.org/packages/clustComp/ and contains a ‘vignette’ outlying the typical use of the algorithms.

**Supplementary information:**

Supplementary data are available at *Bioinformatics* online.

## 1 Introduction

Clustering is an exploratory, unsupervised technique commonly used in the analysis of gene expression data to gain biological insight at genomic level. However, there is no universal method that is appropriate for all uses. A wide variety of clustering algorithms has been developed for gene expression data in the past. For a revision of methods applied to this type of data, see, e.g. Pirim *et al.* (2012). Different outputs are often difficult to compare, and though there exists a number of techniques to assess the agreement between two clustering outputs, either from flat (Hubert and Arabie, 1985; Pinto *et al.*, 2008) or hierarchical (Fowlkes and Mallows, 1983; Perotti *et al.*, 2015) clusterings, they do not establish a mapping between the clusters of each partition. This would be useful, for instance, for understanding the accuracy of a discriminant analysis for tumour classification, as it would help visualize the performance of a classifier in terms of the known groups.

In this note, we introduce the clustComp package, part of the Bioconductor project (Gentleman *et al.*, 2004). By constructing a mapping between groups of clusters, referred to as superclusters, it implements different techniques for the comparison and visualization of relationships between clustering results, either flat versus flat or hierarchical versus flat.

## 2 Methods and implementation

The package addresses the problem of identifying relationships between two different gene expression clustering outputs. The simplest situation corresponds to the comparison of two flat clusterings $A=\{A_{1},\ldots ,A_{m}\}$ and $B=\{B_{1},\ldots ,B_{n}\}$. First, for each pair of clusters (*A_i_*, *B_j_*) their intersection $A_{i}\cap B_{j}$ is computed; then a greedy algorithm maps each cluster *A_i_* with the cluster(s) from B having the largest intersection with *A_i_*, and analogously with each cluster *B_j_*. If more than a cluster on one side is mapped to the same cluster(s) on the other side, they are merged into superclusters to produce a one-to-one mapping.

Such mapping and the contribution of each cluster to the superclusters is visualized using a weighted bipartite graph. Nodes on each layer of the graph represent clusters from each clustering, with the same labels. An edge connects a pair of nodes *A_i_* and *B_j_* if the intersection between the associated clusters is non-empty and the weight assigned to this edge is given by the cardinality of $A_{i}\cap B_{j}$. Thus, edges are drawn with thickness proportional to their weight. The best layout for the graph is defined in terms of the number of weighted-edge crossings. To minimize this number, an NP-hard problem (Garey and Johnson, 1983), we reorder the nodes on each layer using the generalization of the barycentre algorithm (Gansner *et al.*, 1993) provided in Torrente *et al.* (2005). To speed up the computation of the number of edge crossings, we have generalized the dynamic programming algorithm developed by Nagamochi and Yamada (2004) to graphs with weighted edges (see the details in the Supplementary Material).

In case of comparing a hierarchical and a non-hierachical clusterings, the graph representation is adapted as follows (Torrente *et al.*, 2005): the flat clustering is displayed on one side as before, while the other side holds a number of collapsed branches from the dendrogram. Starting at the root, the tree is explored by depth-first search to decide at each step if the branch under consideration should be split or pruned. The decision is made using one of two possible scoring functions that compare the graph having the branch collapsed with that obtained after expanding it. The first scoring function is based on the aesthetics of the graph as it allows expanding branches if this produces few thick edges rather than many small edges, and penalizes the formation of many new crossings. The second scoring function codifies the information about one clustering contained in the other by means of conditional probabilities; in this case a splitting will take place if describing one clustering in terms of the other requires less bits, on average, in the case of replacing the branch with its descendants. Though branches are represented in the same way as flat clusters, the barycentre algorithm can only be used on the flat layer; however, in order to decrease the number of edge crossings, two consecutive branches can swap their positions if they are the descendants of a common branch.

The package contains two basic functions, flatVSflat and flatVShier, to perform the comparisons and to display the best graph layout. They include several parameters, which are standard R objects that give flexibility with respect to the analysis and the visualization. The outputs include vectors indicating the supercluster each gene is allocated to, as well as a description of how initial clusters are arranged into superclusters. Therefore, they can be reused in further analyses. The Supplementary Material and the user documentation provide additional features about the utilization of these and related functions.

## 3 Application

To delineate the performance of the package we used a real RNA-seq dataset, derived from ArrayExpress experiment E-GEOD-30352 (Brawand *et al.*, 2011). This contains 21 human samples from five different tissues of origin. After appropriate preprocessing of the data, we selected, for illustration purposes, the 100 most variable genes and centred them across samples (refer to the Supplementary Material for further details).

**Fig. 1:**
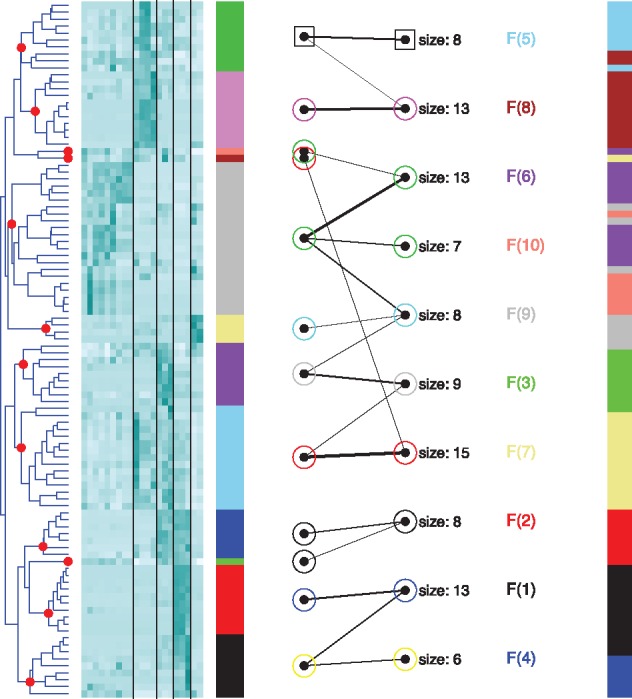
Comparison of a hierarchical clustering and a flat clustering, using the scoring function based on the aesthetics of the graph. The comparison produces twelve branches in the dendrogram, most of them corresponding to genes overexpressed in one or two tissues (delimited by vertical black lines). When applying the greedy algorithm, branches and flat clusters are combined into nine superclusters, identified with coloured symbols at the nodes

As an example, we produced a hierarchical tree and a flat clustering, using respectively complete linkage and k-means with ten clusters, both with Euclidean distance. We compared them using the aesthetics-based scoring function. Figure 1, where gene labels have been removed due to space restrictions, displays the most detailed visualization of the comparison provided in the package. More compact versions, suitable for large datasets, are shown in the Supplementary Material. The dendrogram cut-offs, at different heights, are indicated with a red dot on the branch to be collapsed, and the sizes of the resulting 12 clusters are visualized with a coloured bar, on the left. The heatmap shows that the branches correspond to groups of genes that are overexpressed in one or two tissues. An additional coloured bar on the right displays how genes are distributed across flat clusters. The greedy algorithm merges the branches and flat clusters into superclusters, and the mapping between them is indicated by labelling the nodes with coloured symbols. Further analyses are considered in the Supplementary Material.

## 4 Discussion

We have developed clustComp, an open source Bioconductor package for the comparison and visualization of relationships between clustering results produced by different algorithms or parameters, to enhance their similarities and differences, or to assess their quality. The implemented techniques are based on the identification of superclusters and on the representation of flat clusters/branches from the dendrogram as nodes in a weighted bi-graph. The package provides flexibility in the visualization, allowing for different versions, which makes it suitable for both small and large datasets. In particular, plots can include or not the heatmap of the data, or can display collapsed or expanded dendrograms. This is specially useful in the case of very large datasets. As the algorithms are solely based on clustering outputs, they are equally applicable to microarray or sequencing data, as illustrated with real datasets.

## Supplementary Material

Supplementary Data
