# Supplementary material for: clustComp, a bioconductor package for the comparison of clustering results
Source: Bioinformatics. 2017 Aug 23;33(24):4001–3. doi: 10.1093/bioinformatics/btx532 (PMC5860092; doi:10.1093/bioinformatics/btx532)
Supplement: Supplementary Data [file btx532_supplementary.pdf]

Supplementary Material to accompany the manuscript 'clustComp, a Bioconductor package for the comparison of clustering results'

Aurora Torrente and Alvis Brazma

1 Dynamic programming algorithm for counting edge crossings

Consider a bipartite (or 2-layered) graph (N, E), with m and n nodes on each side, connected by weighted edges. Denote by $V = \{v_1, \ldots, v_m\}$ and $W = \{w_1, \ldots, w_n\}$ the two disjoint sets of nodes, with $N = V \cup W$. If all edge weights are non-negative integers, an edge $e_{ij} = (v_i, w_j)$ with weight $weight_{i,j}$ can be considered as $weight_{i,j}$ unweighted edges connecting the same pair of nodes (i.e., as multiple edges). Assume that for a particular graph layout the vertices in V are ordered from bottom upwards and those in W are ordered from top downwards (see Figure 1). Also, let us denote $V_i = \{v_1, \ldots, v_i\}$, $i = 1, \ldots, m$, and $W_j = \{w_1, \ldots, w_j\}$, $j = 1, \ldots, n$, with $V_0 = W_0 = \emptyset$. The number of edges connecting each pair of vertices (v_i, w_j) is given by the entry (i, j) of the matrix $weight = [weight_{ij}]_{m\times n}$.

The following procedure generalises the algorithm described in [1] to the case of multi-edge bipartite graphs, and allows computing the number of crossings in the given layout. At each step, we count the number of crossings created by the edge e_{ij} and every other edge connecting nodes from V_{i-1} and W_{j-1}.

procedure $countCrossing(weight)$
  input: $weight$, an $m \times n$ matrix.
  $edges := [0]_{m\times n}$
  $crossings := 0$
  for $i := 2, \ldots, m$ **do**
    for $j := 2, \ldots, n$ **do**
      $edges_{ij} := edges_{i,j-1} + edges_{i-1,j} - edges_{i-1,j-1} + weight_{i-1,j-1}$
      $crossings := crossings + edges_{ij} * weight_{ij}$
    end for
  end for
  output: $crossings$

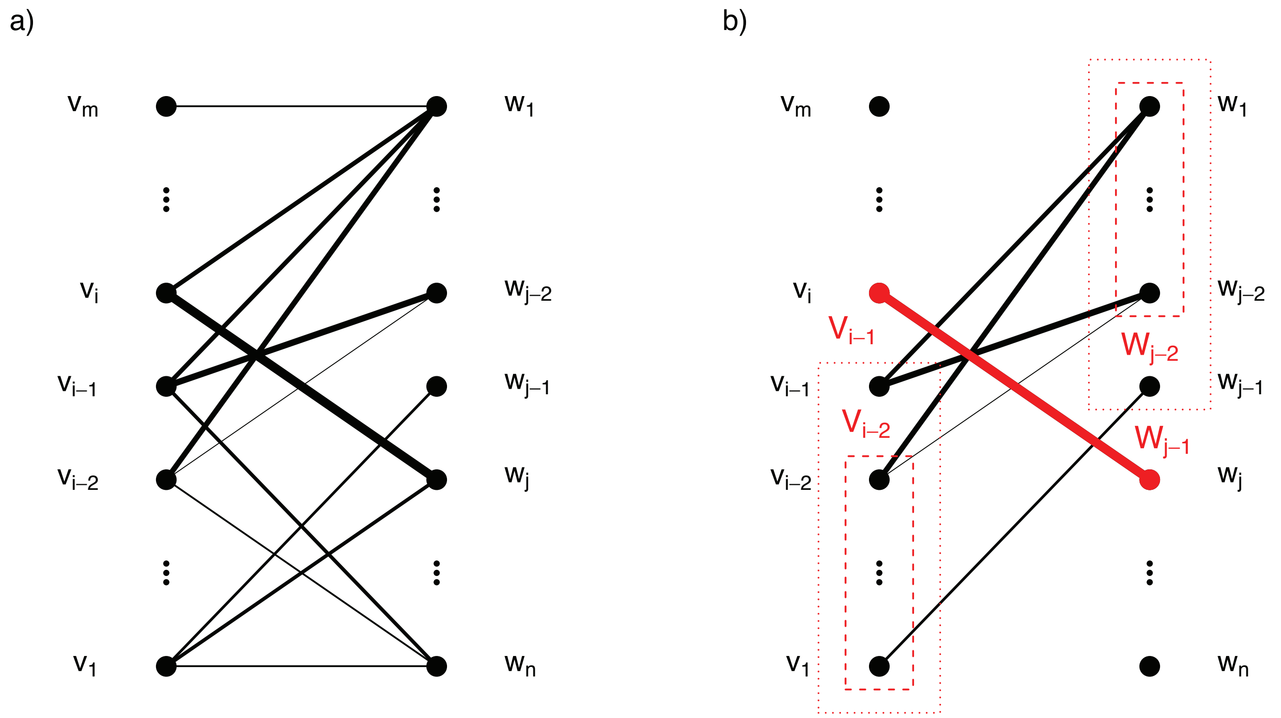

Figure 1: a) A 2-layered layout for a weighted, bipartite graph with sets of vertices $V = \{v_1, \ldots, v_m\}$ and $W = \{w_1, \ldots, w_n\}$. The edge thickness represents the number of multiple edges connecting each pair of nodes. b) The subsets of vertices required to count, in a dynamic-programming way, the edge crossings created by edge (v_i, w_j) are displayed in red.

2 Results

We provide extensive details of the different comparisons that can be performed with the package.

2.1 RNA-seq data

We first consider the RNA-seq dataset used in the accompanying manuscript, derived from the dataset in [2]. It contains the expression data corresponding to 21 samples from healthy brain, heart, kidney, liver and testis. We considered the 62757 genes common to all samples. Counts were transformed into RPKM and genes with more than 30% of zeros were discarded. For illustration purposes, we selected the 100 most variable ones and centred them across samples.

We used `hclust` and `kmeans`, as implemented in the R `base` package, both with Euclidean distance, to obtain respectively a complete linkage hierarchical clustering and different flat partitions of the genes.

Flat vs flat

We obtained two flat clusterings with ten and seven clusters, stored as vectors `flat1` and `flat2`. First we consider the barycentre algorithm, which finds the optimal graph layout in terms of edge crossings. A call to the function `flatVSflat` of the form:

```
> flatVSflat(flat1, flat2, evenly=TRUE, greedy=FALSE)
```

produces the reordered bi-graph shown in Figure 2.a) and outputs the initial and final number of edge crossings, as well as the coordinates to represent the optimal layout. Here, the number of crossings has been reduced to zero. The argument `evenly` is used to display the nodes equally spaced along the Y axis.

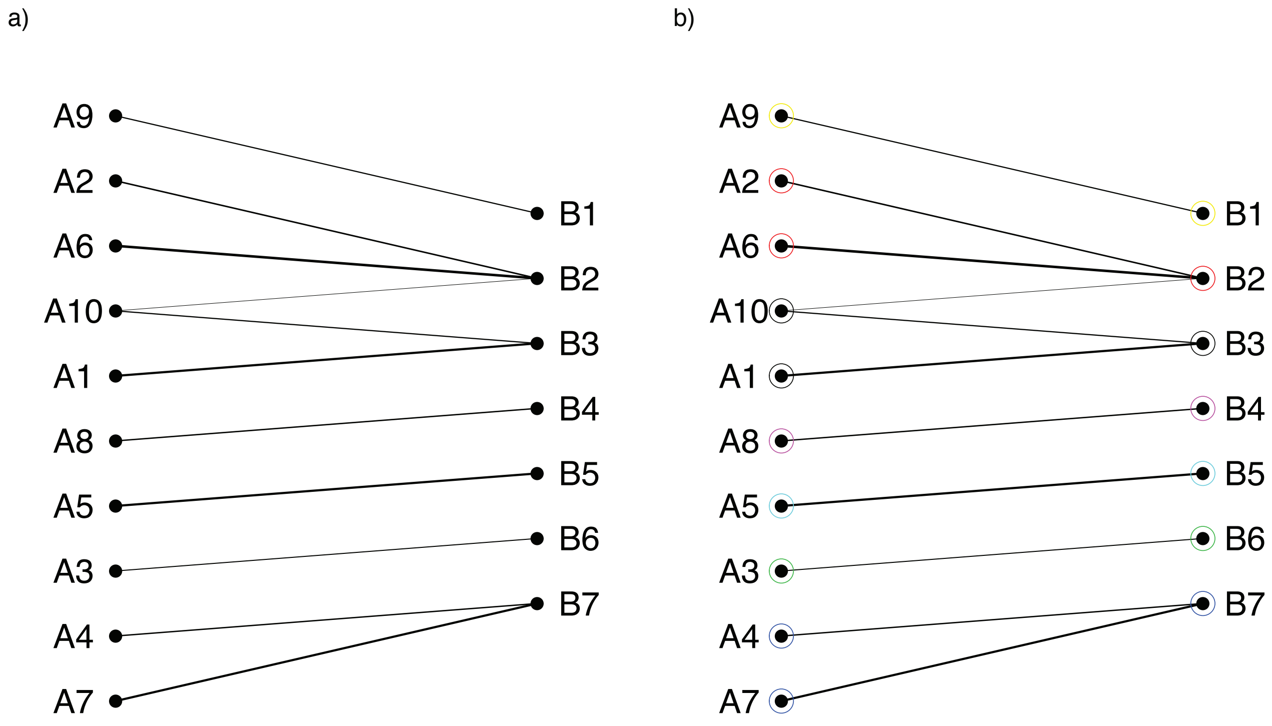

Figure 2: Comparison of two flat clusterings with ten and seven clusters. a) This graph layout, obtained with the generalised barycentre algorithm, sets the number of crossings to 0. b) Superclusters are identified in terms of the elements shared by flat clusters. All superclusters on the right contain only one cluster.

If in addition we desire to display the superclusters found by the greedy algorithm we can use:

```
> flatVSflat(flat1, flat2, even=TRUE, greedy=TRUE)
```

and get coloured symbols to identify them (see Figure 2.b). Both partitions are very similar and thus only three pairs of clusters from the first partitioning are collapsed. In this situation, the output of the function also includes information on how initial clusters are merged to form superclusters and the distribution of genes across them. A more compact visualization of the one-to-one mapping between the superclusters can be obtain with a direct call to the internal function `SCmapping` of the form:

```
> SCmapping(flat1, flat2)
```

This function maps the superclusters as in Figure 3, and indicates how original clusters are merged in the one-to-one mapping.

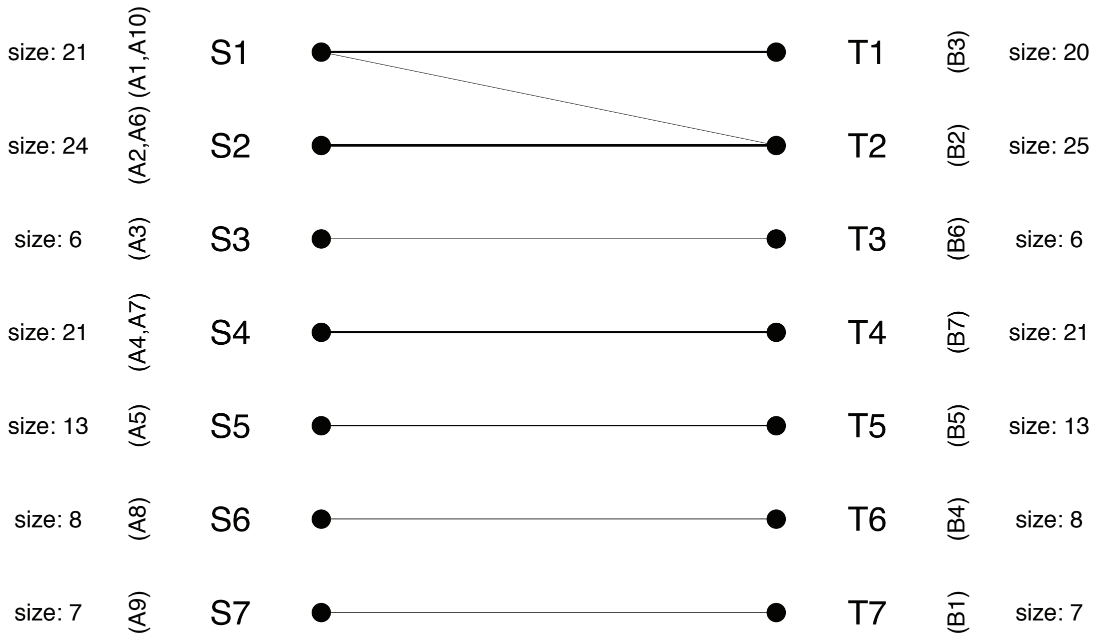

Figure 3: Compact visualization of the mapping among superclusters, including their size.

Hierarchical vs flat

Next we compared the flat clustering with ten clusters from previous paragraph and a hierarchical tree using complete linkage and Euclidean distance. Assume the corresponding `hclust` object is stored as `hierar` and that the gene expression data are given by matrix `RPKM`. Figure 4 shows the same visualisation as that in the accompanying manuscript, with the corresponding gene labels, produced by:

```
> flatVShier(hierar, flat1, expanded=TRUE, expression=RPKM, greedy=TRUE,
     score.function='crossing', look.ahead=0)
```

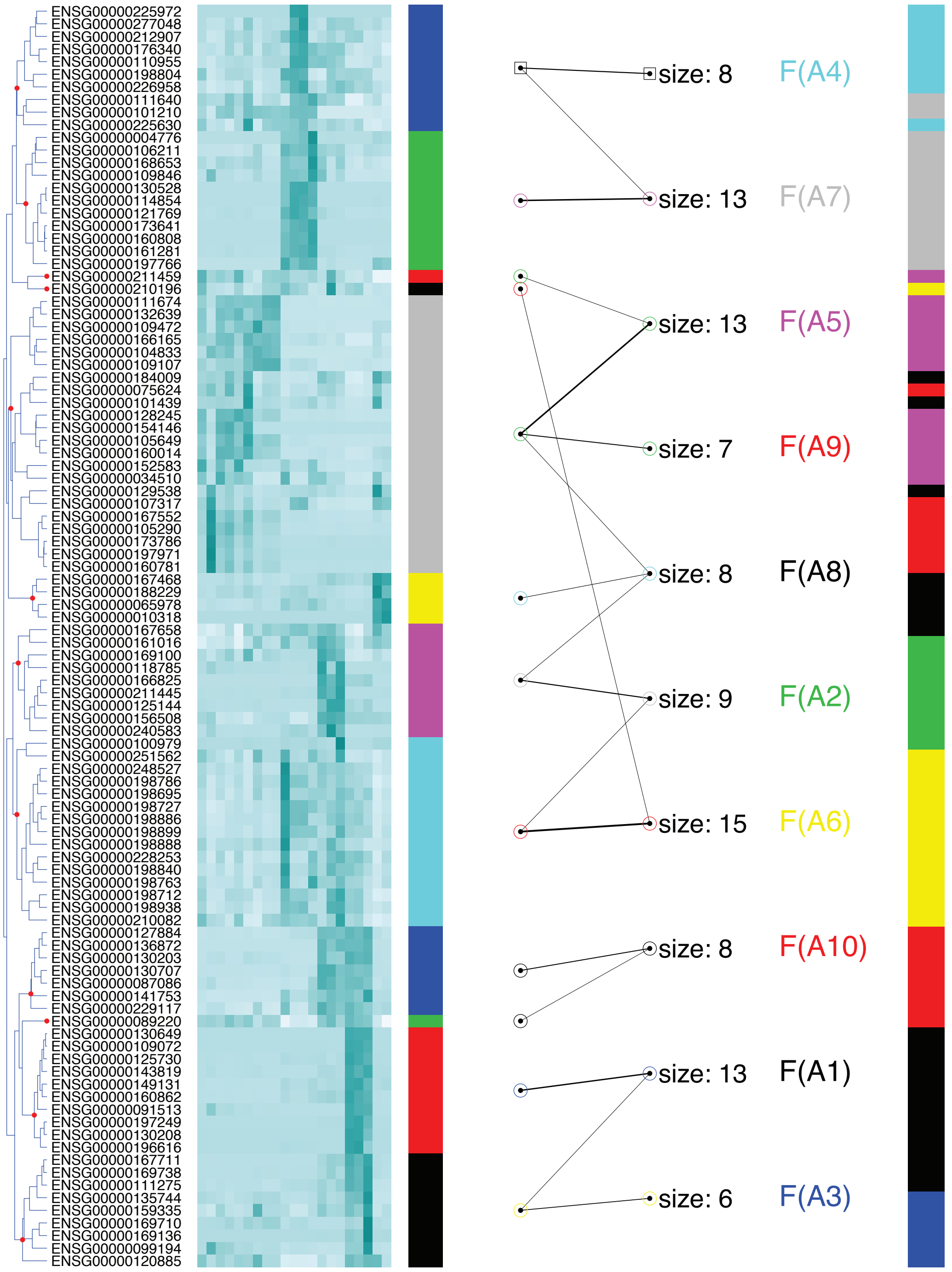

Figure 4: Comparison of a hierarchical tree and a flat clustering with ten clusters using the aesthetics-based scoring function. The dendrogram is expanded to show all the leaves in the pruned branches. The heatmap is included to provide full information on the expression levels. The dendrogram is explored without looking ahead.

Parameters `expanded` and `expression` control the appearance of the plot, by forcing the dendrogram to be fully displayed and by including the heatmap of the data, respectively. The parameter `greedy` allows labelling the superclusters like in the flat versus flat case. The other two arguments, `score.function` and `look.ahead`, modify the analysis in the following way.

When `score.function` is set to `"crossing"`, the method to decide whether a given branch in the dendrogram has to be split or collapsed is based on the aesthetics of the resulting graph, as it is intended to minimise the number of new crossings by finding a compromise between this number and the formation of few thick edges rather than many thin ones. The exact formula for this function is given in [3]. A different criterion to explore and prune the dendrogram is given by an information theoretic scoring function, which is based on the mutual information shared by the two clusterings. This is a more stringent rule that tends to allow less splits than the aesthetics-based one.

The parameter `look.ahead` is an additional feature that allows exploring the dendrogram a given number of generations below a branch whose split is not permitted; thus it is possible to identify appropriate sub-branches that would not be revealed without looking ahead. In Figure 4, the parameter `look.ahead` is set to 0: each time a branch is decided not to be split, there is no further analysis of the descendants of such branch. On the other hand, in the case we were interested in considering the possibility that the score obtained for subsequent generations manages to improve that of the parent branch, we would customise the parameter to look ahead one or more steps, in the form:

```
> flatVShier(hierar, flat1, expanded=TRUE, expression=RPKM, greedy=TRUE,
      score.function='crossing', look.ahead=1)
```

(see Figure 5). This allows a better matching between the two partitionings, as enhanced by the coloured bar on the right.

Setting the parameter `score.function` to `"it"` produces, without looking ahead, less branches in the dendrogram, as expected; see Figure 6. The different visualisation options can be combined to produce more compact plots, a useful aspect in the case of dealing with large datasets. For instance, the version displayed in Figure 6.a), is obtained with the command:

```
> flatVShier(hierar, flat1, expanded=FALSE, greedy=TRUE, score.function='it',
      look.ahead=0)
```

A text label is appended to reflect the size of the flattened branches. On the other hand, the look-ahead strategy

```
> flatVShier(hierar, flat1, expanded=FALSE, greedy=TRUE, score.function='it',
      look.ahead=1)
```

allows relocating some samples from the tree in different superclusters, as shown in Figure 6.b).

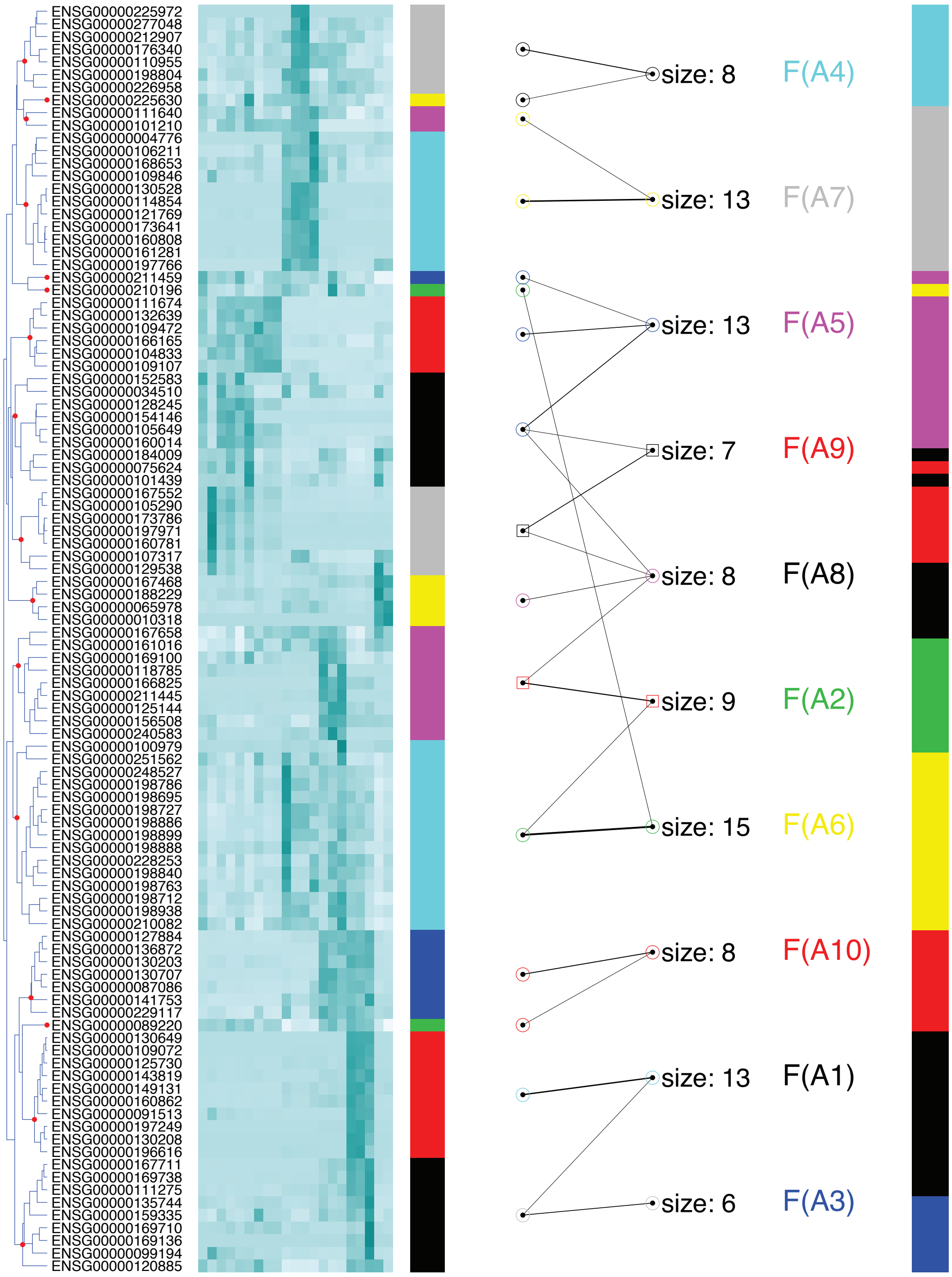

Figure 5: Comparison of a hierarchical tree and a flat clustering with ten clusters using the aesthetics-based scoring function. The dendrogram is expanded to show all the leaves in the pruned branches. The heatmap is included to provide full information on the expression levels. The tree is searched further, by looking one step ahead at each iteration. This allows obtaining more branches.

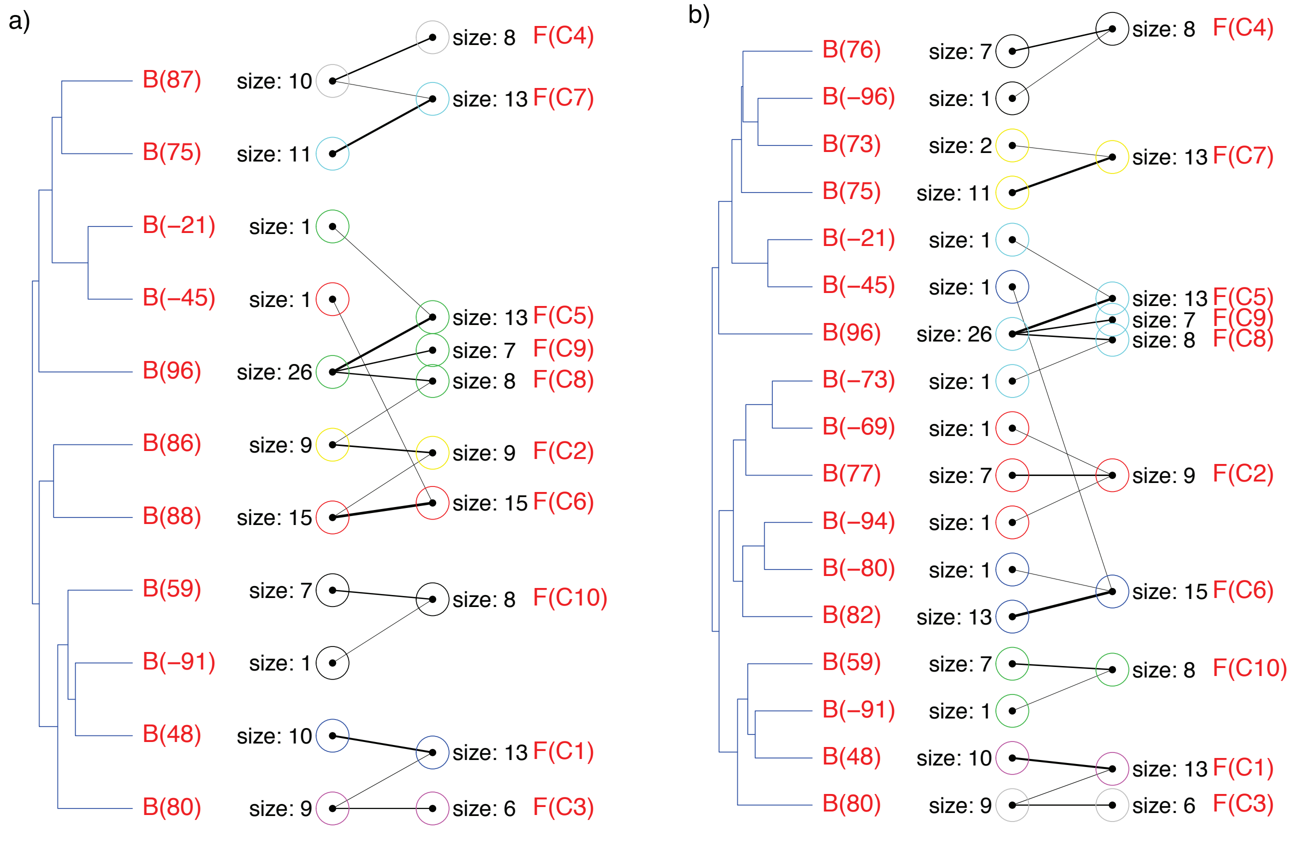

Figure 6: Comparison of a hierarchical tree and a flat clustering with ten clusters, using the information theoretic scoring function. The dendrogram is collapsed at the pruned branches: a) not looking ahead; b) looking ahead one step.

2.2 Microarray data

It is worthy remarking that the techniques implemented in the package can be applied to any data type, as they only use clustering outputs as arguments. Thus, we further illustrate the performance of the methods on the breast cancer microarray data described in [4], which were preprocessed and filtered as in [5]. The dataset is available at http://www.broadinstitute.org/cgi-bin/cancer/datasets.cgi, and contains the expression level of 1213 genes across 49 samples from 4 different types of tumours. All the clustering techniques are applied to samples.

Flat vs flat

For this dataset the true labels of each sample are known, and thus the comparison can be used to assess the suitability of a given clustering method. For instance, Figure 7.a) compares a clustering with four groups and the four real classes; clearly, k-means performs poorly at identifying four groups of samples. A call to the function `SCmapping` shows that the algorithm

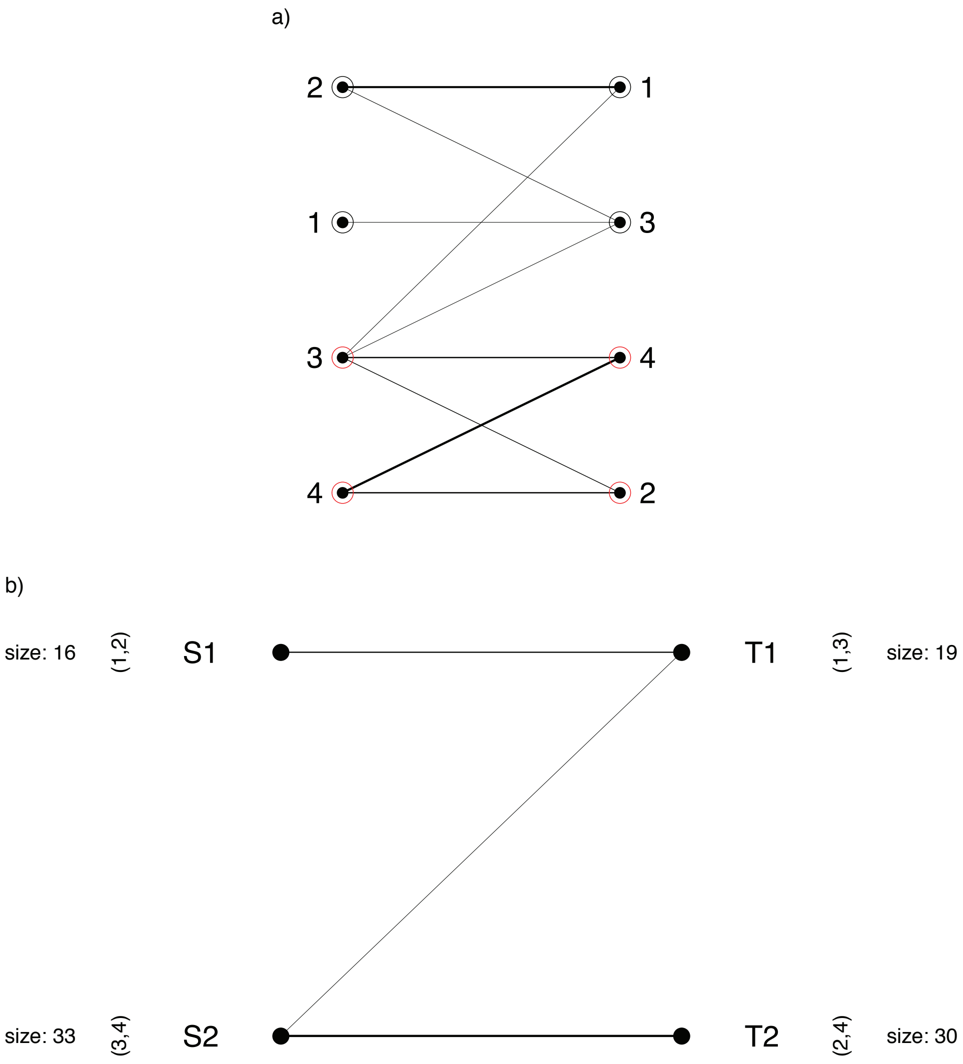

Figure 7: Comparison of a flat clustering (using k-means with four clusters), on the left, and the true partitioning of the samples, on the right. a) k-means does not recover true groups correctly. b) The greedy algorithm illustrates how true groups have been merged with k-means.

puts true groups 1 and 3, and 2 and 4 together, but the resulting superclusters still overlap. See Figure 7.b).

With respect to the comparison of two proper flat clustering results, we first considered two partitions with four and six clusters, respectively. We ran the algorithm with different initial points to produce different outputs. The top panels of Figure 8 show a trivial situation, where one of the clusterings is a subpartition of the other and mapping groups of clusters from both partitions is straight forward (panel a). The superclusters from both clusterings are identical and thus they do not exhibit any overlap (panel b). The bottom panels of Figure 8 correspond

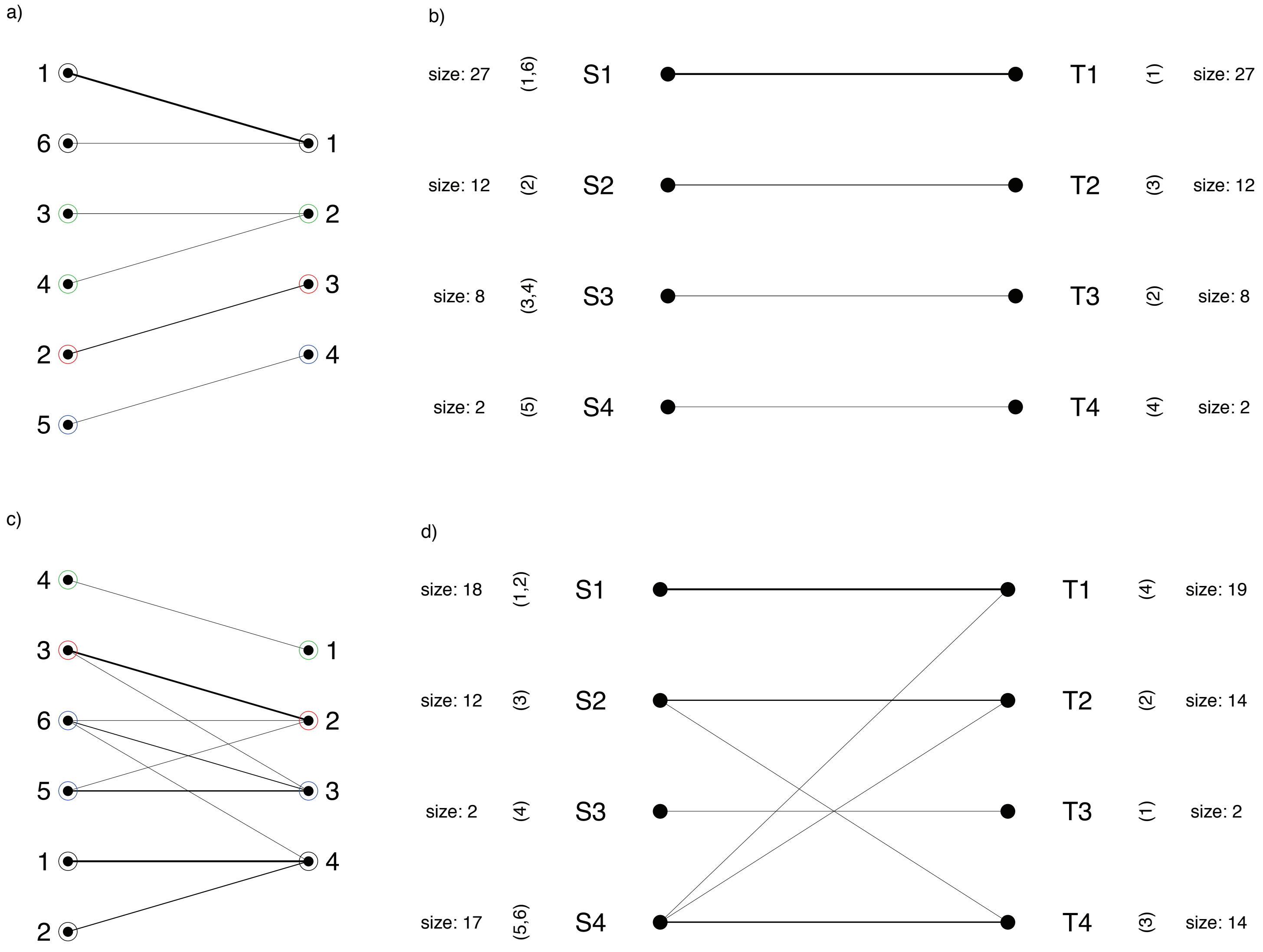

Figure 8: Comparison of two flat clusterings with six (left) and four (right) clusters, using k-means. a) Trivial comparison when one of the partitions is finer than the other: the clusters on the right are subsets of those on the left. b) The greedy algorithm identifies and preserves the four clusters from the four-cluster partitioning, setting a one-to-one mapping between identical superclusters (with no overlaps). c) Non-trivial comparison when none of the partitions contains the other. d) Some of the superclusters found by the greedy algorithm do overlap.

to partitions where none is included in the other; the barycentre algorithm helps visualise the similarities by reducing the number of crossings from the initial 583 to 17 (panel c), whereas the greedy algorithm identifies three out of four pairs of non-identical superclusters (panel d). Note that the visualisation of the function `SCmapping` is not designed to minimise the edge

crossings but to display one-to-one relationships, provided by the horizontal edges.

Hierarchical vs flat

We also compared a hierarchical tree (complete linkage, Euclidean distance) to a flat partitioning with six clusters (k-means, Euclidean distance). As in the case of flat clusters, the dendrogram does not accurately reflect the true sample groups; nevertheless, it can be compared to a non-hierarchical clustering to enhance similarities or differences between the clustering results.

First we considered the scoring function based on the aesthetics of the graph. As previously mentioned, this is in general a strategy less stringent than the information theoretic one, as it tends to allow more branch splittings (see [3]). When no look-ahead is used two clusters from the flat side are merged into one supercluster, but the structure provided by the rest of groups is similar to that found in the tree, as shown in Figure 9.a). On the other hand, by looking one step ahead, it is possible to preserve each cluster from the right layer of the bi-graph: the greedy algorithm finds a one-to-one correspondence between flat clusters and groups of branches (see Figure 9.b)).

Finally, we considered in Figures 9.c) and d) the comparison using the information theoretic score. In this case, it is harder to identify similarities between the clusterings: in a first attempt without looking ahead, no agreement is essentially found. However, searching the tree one step ahead improves the situation and two "large" superclusters are identified on each side of the graph.

References

[1] Nagamochi,H. and Yamada,N. (2004) Counting edge crossings in a 2-layered drawing, *Inform Process Lett*, **91**, 221–225.

[2] Brawand,D. *et al.* (2011) The evolution of gene expression levels in mammalian organs, *Nature*, **478**(7369), 343–348.

[3] Torrente,A. *et al.* (2005) A new algorithm for comparing and visualizing relationships between hierarchical and flat gene expression data clusterings, *Bioinformatics*, **21**(21), 3993–3999.

[4] West,M. *et al.* (2001) Predicting the clinical status of human breast cancer by using gene expression profiles. *Proc Natl Acad Sci USA*, **98**, 11462–11467.

[5] Hoshida,Y. *et al.* (2007) Subclass Mapping: Identifying Common Subtypes in Independent Disease Data Sets. *PLOS One*, **2(11)**, e1195.

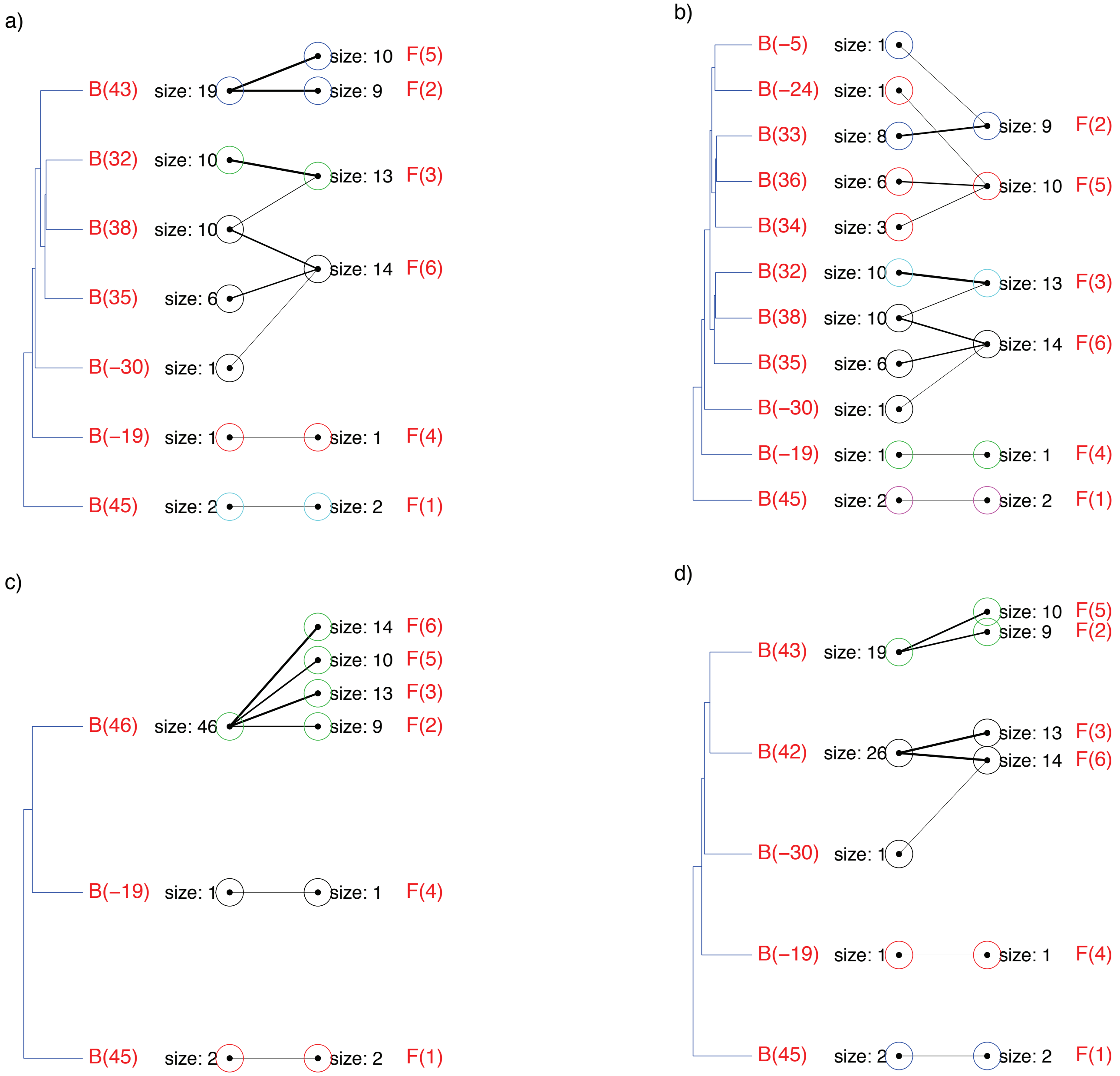

Figure 9: Comparison of a hierarchical clustering (complete linkage), on the left, and a flat clustering (k-means, six clusters), on the right. a) The scoring function is the one based on the aesthetics of the graph; no look-ahead is used. Though two flat clusters are combined into a supercluster, the comparison enhances a noticeable similarity between both clusterings. b) Aesthetics-based scoring function, looking one step ahead. In this case, the six clusters are preserved when applying the greedy algorithm. c) Information theoretic scoring function; no look-ahead. Nearly no similarities are found with this stringent criterion, and all the “large” flat clusters are collapsed into one. d) Information theoretic scoring function; looking one step ahead. More similarities are identified, but still less than with the aesthetics-based function.
